# Characterization and Evaluation of Antimicrobial Potential of *Trigonella incise* (Linn) Mediated Biosynthesized Silver Nanoparticles

**DOI:** 10.3390/molecules27144618

**Published:** 2022-07-20

**Authors:** Fozia Fozia, Nisar Ahmad, Zohra Aftab Buoharee, Ijaz Ahmad, Madeeha Aslam, Abdul Wahab, Riaz Ullah, Shakeel Ahmad, Amal Alotaibi, Akash Tariq

**Affiliations:** 1 Boichemistry Department, Khyber Medical University Institute of Medical Sciences, Kohat 26000, Pakistan; drfoziazeb@yahoo.com; 2 Department of Botanical & Environmental Sciences, Kohat University of Science & Technology, Kohat 26000, Pakistan; ahmed_kust@yahoo.com (N.A.); ziohrabotanist44@gmail.com (Z.A.B.); 3 Department of Chemistry, Kohat University of Science & Technology, Kohat 26000, Pakistan; drijaz_chem@yahoo.com (I.A.); madeehaaslam06@gmail.com (M.A.); saislamian@yahoo.com (S.A.); 4 Department of Pharmacy, Kohat University of Science & Technology, Kohat 26000, Pakistan; wahabscholar@yahoo.com; 5 Department of Pharmacognosy (MAPPRC), College of Pharmacy, King Saud University, Riyadh 11451, Saudi Arabia; 6 Department of Basic Science, College of Medicine, Princess Nourah Bint Abdulrahman University, P.O. Box 84428, Riyadh 11671, Saudi Arabia; amaalotaibi@pnu.edu.sa; 7 Xinjiang Key Laboratory of Desert Plant Roots Ecology and Vegetation Restoration, Xinjiang Institute of Ecology and Geography, Chinese Academy of Sciences, Urumqi 830011, China; akash.malik786@mails.ucas.ac.cn

**Keywords:** *Trigonella incise*, green synthesis, silver nanoparticles, drug sighting

## Abstract

The goal of the research was to explore a new green method used to synthesize silver nanoparticles (Ag NPs) from an aqueous extract of *Trigonella incise*, which serves as a reducing and stabilizing agent. The obtained results showed an 85% yield of nanoparticles by using 2:5 (*v*/*v*) of 5% plant extract with a 0.5 M solution of AgNO_3_. Different techniques were used to characterize the synthesized Ag NPs, including X-ray diffraction (XRD), Fourier-transform infrared spectroscopy (FT-IR), scanning electron microscopy (SEM), and UV–visible spectroscopy. The UV–visible spectra of green synthesized silver nanoparticles showed maximum absorption at a wavelength of 440 nm. The FT-IR studies revealed the stretching oscillation frequency of synthesized silver nanoparticles in the absorption band near 860 cm^−1^. Similarly, the bending and stretching oscillation frequencies of the NH function group were assigned to the band in the 3226 cm^−1^ and 1647 cm^−1^ regions. The bending vibration of C-O at 1159 cm^−1^ confirmed the carbonyl functional group that was also assigned to the small intensity band in the range of 2361 cm^−1^. The X-ray diffraction analysis of Ag NPs revealed four distinct diffraction peaks at 2θ of 38°, 45°, 65° and 78°, corresponds to (111), (200), (220) and (311) of the face-centered cubic shape. The round shape morphology of Ag NPs with a mean diameter in the range 20–80 nm was analyzed via SEM images. Furthermore, the nanoparticles showed more significant antimicrobial activity against *Salmonella typhi* (*S. typhi*) and *Staphylococcus aureus* (*S. aureus)* with an inhibition zone of 21.5 mm and 20.5 mm at 6 μg/mL concentrations, respectively, once compared to the standard reference. At concentrations of 2 µg/mL and 4 µg/mL, all of the bacterial strains showed moderate activity, with inhibition zones ranging from 11 mm to 18.5 mm. Even at high concentrations of AgNPs, *S. typhi* showed maximum resistance. The best antifungal activity was observed by synthesized Ag NPs against *Candida albicans* (*C. albicans*) with 21 mm zone of inhibition, as compared to a standard drug which gives 22 mm of inhibition. Therefore, we conclude that the antibacterial and antifungal activities showed satisfactory results from the synthesized Ag NPs.

## 1. Introduction

Nanotechnology frameworks are used to describe a wide range of commercially viable and pharmaceuticals polymers. Various metallic nanoparticles are used for the synthesis through a green approach, including gold, silver, copper, zinc, and titanium. Metallic nanoparticles have a high fraction of surface atoms and a large surface area, making them suitable for various application [1]. Due to their exclusive properties, metallic nanoparticles are widely used in various fields such as clothing, cosmetics, and medicine, as well as energy production in oxides and solar fuel batteries. Among various metallic nanoparticles, Ag NPs have received quite a bit attention and are the most widely used metal nanoparticles because of their unique assets, such as size, shape and reliance on optical, magnetic, and electrical properties [2]. Silver (Ag) is a soft, white, lustrous metal that shows good electrical and thermal conductivity and has a special significance in terms of medical and therapeutic benefits which outweigh the risks [3]. The physiochemical and biological approaches of using Ag NPs have sparked a lot of interest to determine catalytic, insecticidal [2], anti-angiogenesis, anti-inflammatory, anti-platelet, and antiviral activities [4]. Silver nanoparticles have been demonstrated as antimicrobial agents for the healing process [5], ointment balms for wound infections [6], anticancer agents [7], and are also used in coating stainless steel products, the treatment of burns, sunscreen lotion, water treatment and dental materials [8,9].

Different chemical, physical, and biological methods have been employed to synthesize Ag NPs. Those approaches include the chemical reduction method [10], pulsed laser deposition [11], the photo reduction method [12], the sono-electrochemical method [13], microwave-induced electrolysis [14], the salvo method, aerosol flow reactors, photochemical reduction, spray pyrolysis, spark discharge and chemical fluid deposition [15]. The chemical approaches result in chemical contamination, which is dangerous to the environment and human health, whereas physical methods produce low yields, and the used of toxic solvents and chemical contamination of nanoparticles restrict their use in biomedical applications. Therefore, the need for environmentally friendly production of Ag NPs has attracted attention in biogenic approaches. Several microorganisms, such as algae [16], bacteria [17] and fungi [18], have been utilized to demonstrate the use of successful synthesis of Ag NPs that reduces chemical expenses and chemical toxicity, and provides easy extraction. On the other hand, plant-based methods employed in Ag NPs synthesis are among the most widely used biological methods, due to their wide distribution, safe handling, ease of availability, and being a good source of metabolites. Several reports on the synthesis of Ag NPs are available, employing various plant extracts such as *Morus indica* [19], *Berberis vulgaris* [20], *Tagetes erecta* [21], which act as stabilizing and reducing agents.

The current study is designed to synthesize Ag NPs by using an aqueous plant extract of *Trigonella incise* which serves as a capping and reducing agent. *Trigonella incisa* Linn is a genus with 70 species and belongs to the Fabaceae family, which is found in the Mediterranean zone. In Pakistan, there are 16 different *Trigonella* species. *Trigonella incisa* is a perennial plant and its leaves are trifoliolate and pinnate. It has branched and prostrate stems, a tape root, and a linear pod with one-to-many seeds. Some of the important phytochemicals present in *Trigonella incise* are trigonelline, alkaloids, flavonoids, tannin, saponins glycosides, and phenolic compounds. The phytochemicals alkaloids, tannins, flavonoids, and phenolic compounds are primarily necessary to synthesize Ag NPs that act as stabilizing and reducing agents. *Trigonella incise* is used as an antiviral, anti-inflammatory and appetite stimulator in traditional medicine [22]. It is essential to formulate beneficial and reliable pharmaceuticals to prevent various pathogen-caused diseases. The plant in the current research has important biological activities as compared to other plant species that aid in the treatment of several diseases in a sophisticated way. Therefore, we chose *Trigonella incise* medicinal plants as an extremely good biological source for treating a variety of diseases and may be a good target for identifying active natural products using activity guide isolation. Further, there is no specific literature available on synthesis of silver nanoparticles employing *Trigonella incisa* Linn aqueous plant extracts. Therefore, the current study was used for the first time to investigate the synthesis of Ag NPs using an aqueous plant extract and to assess their antimicrobial activities. Furthermore, the synthesized Ag NPs were investigated morphologically, structurally, and optically through various techniques, such as UV–Vis spectrophotometry, SEM, X-ray diffractometer, and Fourier-transform infrared spectroscopy (FT-IR). Moreover, we used different fungal strains and Gram-positive and Gram-negative bacteria to examined the inhibitory activity of synthesized Ag NPs.

## 2. Materials and Methods

### 2.1. Reagents and Chemicals

MERK (Darmstadt, Germany) supplied silver nitrate, which was used as a silver precursor. Sigma Aldrich provided analytical grade methanol and ethanol which was used without further purification. DBH (Darmstadt, Germany) provided nutrient agar and potato dextrose agar media (SDA).

### 2.2. Plant Used

The formation of silver nanoparticles was achieved by employing *Trigonella incise* aqueous plant extracts that served as reducing and stabilizing agents.

### 2.3. Plant Extraction

The whole plant (*Trigonella incise*) was obtained from various marshlands in District Karak and authenticated at the Department of Environmental and Botanical Sciences, Kohat University of Science & Technology, Kohat, Pakistan, wherein a voucher specimen No. DEBS-KUST -7316 was preserved in the herbarium. The collected plant was thoroughly washed with distilled water in order to eliminate the particulates. The wet plant was covered in filter paper and allowed to dry for 15 days in the shed. In a grinder, the dried plant was blended into a fine powder. A fine powder of 100 g was obtained. Then, 25 g of powder plant was mixed with beaker containing 500 mL of distilled water to prepared 5% of plant extract solution. After that, the reaction was heated for 30 min at temperature of 50 °C. The mixture was filtered via Whatman No. 1 (filter paper), and at 3000 rpm, centrifuge was used to separate the crude product. The extract was used at different ratios for the formation of silver nanoparticles.

### 2.4. Synthesis of AgNPs

The synthesis of AgNPs was accomplished by thoroughly mixing 0.5 M silver nitrate solution with plant extract (5%) solution in the following ratios: 1:5, 1.5:5, 2:5, and 2.5:5 (*v*/*v*). After mixing the Ag^+^ solution into an aqueous plant extract, it was placed on a rotatory orbital shaker operating at 200 rpm for 1 h, at 70 °C (adjust the ionic strength of the sample solution to pH 8). After that, to check the availability of the reaction the UV–visible spectroscopy was used. For 5 min, the solution was centrifuged at 3000 rpm. The solvent was eliminated from the residue, which was then again washed with distilled water. The nanoparticles were obtained in powder form, which was used for additional characterization and antimicrobial testing. In addition, a descriptive analysis was conducted to check the influence of various factors including Ag^+^ ion concentration, plant extract volume in the reaction, and reaction time. The UV–Vis spectroscopic analysis was employed to record the reaction mixtures at various intervals. The effect of the metal salts was determined through altering the concentrations of silver nitrate (0.1, 0.2, 0.3, 0.4, and 0.5 M). The influence of time on Ag NP synthesis was ascertained by various intervals of time such as 15, 30, 45, and 60 min. Different volume ratios were used to evaluate the impact of volume ratios on Ag NPs synthesis [23].

### 2.5. Characterization

Characterization of the AgNPs was investigated through several techniques including X-ray diffraction, scanning electron microscopy, Fourier-transform infrared spectroscopy, and UV–visible spectroscopy.

#### 2.5.1. UV–Visible Spectroscopy

The Shimazu double beam UV–visible 1800 spectrophotometer with quartz covet was used for ultraviolet and visible spectroscopic analysis. Then, 1 mL of AgNPs was dispersed in 100 mL of distilled water to evaluate the absorption spectra within the range of 250–800 nm against distilled water as a reference.

#### 2.5.2. FT-IR Analysis

An FT-IR (Furkin-Elmer LS-55-Lumminiscence) spectrometer was used to record the analysis of various functional groups, stabilization and reduction of silver nitrate to Ag NPs. The plant extract preparation for FT-IR spectroscopy involves the use of moderate heat during extraction process. An aqueous extract of plant is poured into a clean container and then placed over water bath or in an oven at a temperature about 50 °C, for 4–8 h. Heat was applied throughout the extraction process to decrease the viscosity of extraction solvent and enhance the removal of secondary metabolites. This method is suitable for preparation of dried extract of plant. After that, the dried extracts of plant were used for FT-IR analysis. Then, 10 mg of the dried extract was encapsulated in 100 mg of KBr pellet, in order to prepare translucent sample discs. The plant sample was loaded in FT-IR spectrophotometer with a scanning range from 400 to 4000 cm^−1^ with a resolution of 4 cm^−1^.

#### 2.5.3. SEM Analysis

The scanning of silver nanoparticles under an electron microscope revealed the distribution and particle diameter of the dehydrated Ag NPs. To achieve the best possible SEM images, certain type of sample requires extra sample preparation. When working with difficult samples, sputter coating can be a useful technique for obtaining high-resolution SEM images. The thickness of sputtered films used in SEM is typically 2–20 nm. The images of the nanoparticles were obtained by sputtering them on the SEM grid.

#### 2.5.4. XRD Analysis

The size, geometry, and crystalline morphology of synthesized nanoparticles were studied using an XRD pattern. The X-ray diffraction pattern has been measured utilizing slump-wrapped film of the sample on a piece of glass and an X-ray diffractometer (INEL DIFFRACTOMETER) with distinctive Cu k1 radiation (=1.78 A) at 0.05/min, within the range of 20° to 90° at constant time (2 s).

### 2.6. Biological Activities

Before assessing the biological effect of Ag NPs against antibacterial and antifungal activity, their stability in culture media, water and buffer solution over 24 h was studied by visible spectroscopy. By performing the reaction, the synthesized Ag NPs remained stable within the concentration range required for biological studies.

#### 2.6.1. Antibacterial Activity

The antibacterial activity of the synthesized nanoparticles was examined utilizing agar well diffusion assay against three bacterial strains: *Staphylococcus aureus*, *Pesudomonas aeruginosa*, *and Salmonella typhi* [24]. The Department of Biotechnology, Kohat University of Science and Technology, Kohat, Pakistan, provided these three bacterial strains: *Salmonella typhi*, *Pseudomonas aeruginosa*, and *Staphylococcus aureus*. The antibacterial activity of Ag NPs was examined by employing different concentrations of silver nanoparticles including 2 µg/mL, 4 µg/mL, and 6 µg/mL. The amoxicillin drug was used as a standard drug for positive control and distilled water, and plant extract was used for negative control. The strains were stored in incubator at 37 °C in nutrient agar media. The standard protocol for nutrient agar media preparation was followed. Approximately 28 g of nutrient agar was dissolved in 1 mL of distilled water. The resultant solution was processed on a hot plate to continuous agitation until it turned into a transparent solution. After that, the solution was enclosed with aluminum foil and preserved in an autoclave set to 1.5-pounds of pressure for 25 min. Following that, each sterilized Petri plate was filled with 25 mL media in a laminar cabinet under an inert atmosphere. The media was solidified at room temperature, and sterile cork borers were used to make the bores. The samples were placed in the appropriate labelled bores. The fortified Petri dishes were incubated for 24 h, at 37 °C. After 24 h, the vernier caliper was used to measure the zone of inhibition [25].

#### 2.6.2. Antifungal Activity

Using a standard protocol agar well diffusion procedure, the antifungal activity of the synthesized Ag NPs was determined against *Fusarium gramium *Alternaria alternata** and *Candida albicans* fungal strains [24]. A similar methodology was used to make Potato Dextrose Broth (PDB). Then, 200 g (peeled) potato were chopped and boiled for 20 min in 100 mL distilled water. The potato extract was mixed with 20 g of Dextrose and 15 g of agar, which was then diluted to 1000 mL distilled water. After adjusting the pH to around 5.6, the solution was incubated for 20 min. The prepared media was allowed to cool before being poured into each Petri plate at about 25 mL. The prepared Petri dishes were labelled and bored with sterile cork borer. The samples of fungal strains were evenly spread over the media. The sample concentrations of 2 µg/mL, 4 µg/mL, and 6 µg/mL were applied to the appropriate labelled bored. The Petri dishes were incubated for 24 h, at 37 °C temperature. After 24 h, the vernier caliper was used to measure the zone of inhibition. The data were then noted in the form of table and graphs. The antifungal drug nystatin was used as a standard [26].

## 3. Results and Discussion

### 3.1. Synthesis of Ag NPs

A green synthetic approach was employed to produce Ag NPs by dissolving a 2:5 (*v*/*v*) ratio of 0.5 M silver precursor and a 5% of plant extract solution and stirring at 60 °C of temperature for 1 h, obtaining an 85% yield of silver nanoparticles. The same result was obtained using the physiological state and optimal ratio. A color transition from light yellow to dark brown indicated the synthesis of silver nanoparticles, which affirmed by UV–visible spectroscopic examination [27].

### 3.2. UV–Visible Spectroscopy

**Figure 1:**
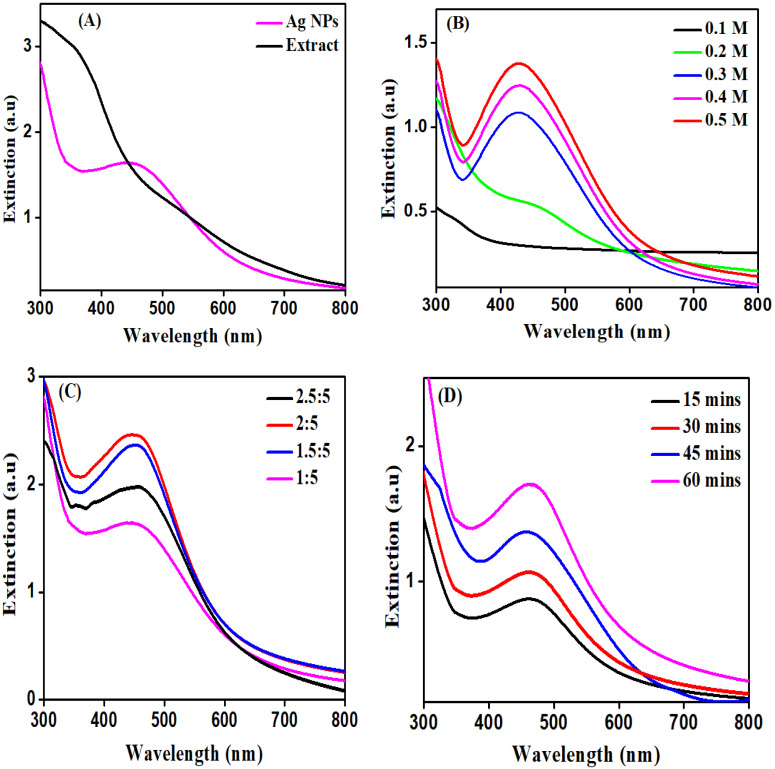
UV–visible analysis of Ag NPs and plant extract (**A**) UV–visible analysis of Ag NPs at different AgNO_3_ concentrations (**B**) UV–visible analysis of Ag NPs at different volume ratio of aqueous silver salt and plant extract (**C**) UV–visible analysis of Ag NPs observed at different time intervals (**D**).

The UV–visible technique was used for the synthesis confirmation of nanoparticles at various wavelengths (200–800 nm) to detect surface plasmon resonance (SPR). The metal nanoparticles provide the surface plasmon resonance band because the conduction band and valence band of metal nanoparticles, such as silver, lie too close to each other, thus photons move freely and vibrate each electron. When the collective oscillation frequency of electrons becomes equal to the incoming wave (UV light) frequency, then strong absorption takes place, which is responsible for the production of surface plasmon resonance (SPR). Figure 1 depicts maximum absorption at wavelength of 440 nm for Ag NPs synthesized by plant extract. The obtained results are consistent with previously reported literature, using the *Spirulina platensis* plant to help to reduce silver ions to silver nanoparticles, and UV–visible absorption showed the SPR (surface plasmon resonance) band for Ag NPs to be at a wavelength of 400–480 nm [28].

For reducing silver ions to Ag NPs using an aqueous plant extract, different factors (such as concentration of silver salt, volume, and reaction time) were used to optimize the formation. The impact of time showed that as the reaction time progresses, the intensity of the surface plasmon resonance bands also increases. As a result, the optimized peak was obtained after 60 min of reaction time, as shown in Figure 1. The UV–Vis absorption spectra, obtained at various AgNO_3_ concentrations, were used to determine the concentration effect on Ag NPs (0.1, 0.2, 0.3, 0.4, and 0.5 M). The result revealed that the SPR band did not appear at the 0.1 M AgNO_3_ concentration, showing insignificant yield of Ag NPs (Figure 1). The best surface plasmon resonance of silver nanoparticles was observed at 440 nm, as the concentration of AgNO_3_ was increased up to 0.5 M, which showed that the peak intensity of the surface plasmon resonance increased remarkably as the concentration of AgNO_3_ was increased. The volume impacts on Ag NP synthesis were evaluated under the required reaction conditions. The findings indicates that by increasing the volume of 1:5. 1.5:5, 2:5, and 2.5:5 (*v*/*v*), the characteristic SPR absorption bands intensity of Ag NPs increases. With further increases in the volume of the reaction, the low intensity SPR peak was obtained [3,29,30].

### 3.3. FT-IR Study

**Figure 2:**
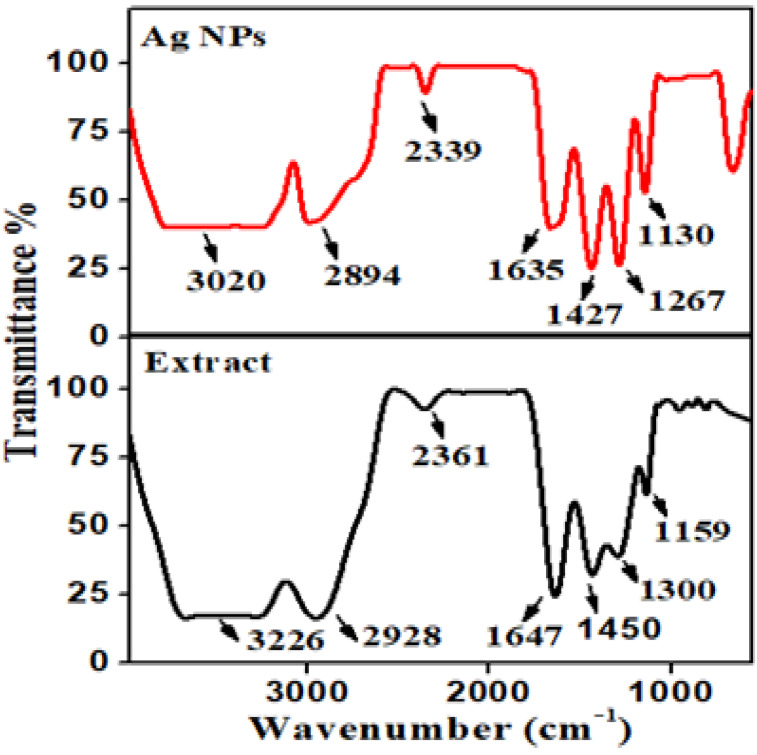
FT-IR combined spectra for Ag NPs and plant extract.

The crucial step in the analysis of medicinal plants is the extraction of the plant, because it is necessary to extract the desired chemical components from the plant. The extraction may be non-polar to polar and thermally labile, so the suitability of the methods of extraction must be considered. The extraction method involves the use of moderate heat during the extraction process. Heat was applied throughout the extraction process and also suitable for the extraction of potential pure active constituents, which are not lost, distorted or destroyed during the preparation of the extract from plant. After that, FT-IR spectrum analysis was performed in order to determine the potential functional molecules bound with silver nanoparticles. The FT-IR spectrum of the plant extract and Ag NPs is shown in Figure 2. The stretching vibration frequency of silver was confirmed by assigning the absorption band near 860 cm^−1^ [31]. The NH function groups stretching vibration was assigned to the broad absorption band in the range 3226 cm^−1^ which was further identified by a band in the 1647 cm^−1^ region. Likewise, the carbonyl functional group was assigned a low-intensity peak in the range of 2361 cm^−1^, and the confirmation of bending vibration of C-O is due to sharp absorption peak at 1159 cm^−1^. The sp_3_ C-H stretching frequency is signified via a prominent band at 2928 cm^−1^. The presence of overtone vibrations between 1300 and 1450 cm^−1^ indicated the existence of aromatic hydrocarbons (Figure 2). The phytochemicals in the plant extract converted Ag^+^ ions to Ag^0^ ions which showed by the reduction in bands at 3020, 2894, 2339, 1635, 1427, 1267, and 1130 cm^−1^. According to FT-IR data, different phytochemicals present in the plant extract possess amino acid, amide, alkaloids, carbonyl, tannins, flavonoids and phenolic hydroxyl with aromatic hydrocarbon. These biomolecules could play a role in reducing silver nitrate to Ag NPs, which are stabilized by the van der Waals electrostatic force and hydrogen bonding [32].

### 3.4. XRD Analysis

**Figure 3:**
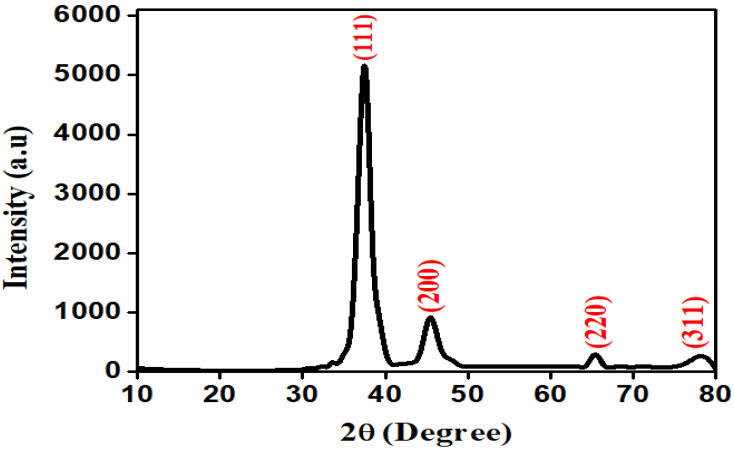
XRD pattern of Ag NPs.

The synthesized Ag NPs revealed four major peaks of diffraction by using an XRD pattern such as (111), (200), (220), and (311), which gives FCC (face-centered cubic) crystalline morphology of Ag NPs at 2θ degree of 38°, 45°, 65°, and 78°. The biosynthesized Ag NPs demonstrate the crystalline nature, which was examined by the highest intensity peak of the XRD pattern as showed in Figure 3. The Debye–Scherrer equation (d = (kλ × 180)/β Cos θ β Π) was used to determine the d-spacing, with 4.32 Å, 2.34 Å, 1.98 Å, and 1.45 Å assigned to 38, 45, 65, and 78 degrees, respectively. As per the Bragg equation, the mean crystallite size was 38.7 nm.

### 3.5. SEM Analysis

**Figure 4:**
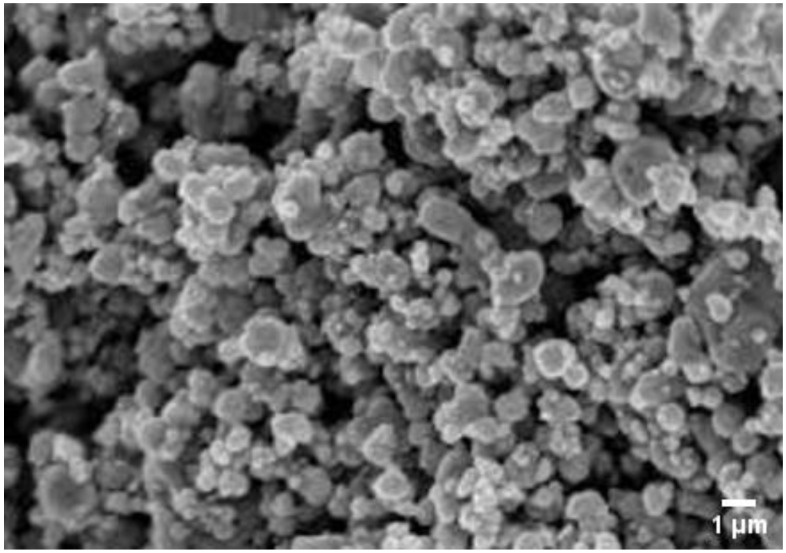
SEM image of silver nanoparticles.

The morphology, shape, and size of silver nanoparticles were examined through SEM images. The nanoparticles were mostly spherical in shape, with mean particle diameter ranges in the range 20–80 nm, as revealed by SEM images (Figure 4). The production of nanoparticles with agglomeration was indicated by highly conglomerate form of nanoparticles. The aggregation of Ag NPs was observed in SEM images, which could be due to the evaporation and removal of solvent used during sample preparation. Solvent removal causes electrostatic forces to bring Ag NPs closer together and aggregated [33].

### 3.6. Antimicrobial Screening

The synthesized Ag NPs exhibited substantial antimicrobial properties against sampling pathogens of human, including *S. aureus*, *P. aeruginosa*, and *S. typhi* bacterial strains and *F. graminearum*, *A. alternata*, and *C. albicans* fungal strain, by using the standard protocol agar well diffusion and drugs (amoxicillin and nystatin) as a standard.

#### 3.6.1. Antibacterial Evaluation

**Figure 5:**
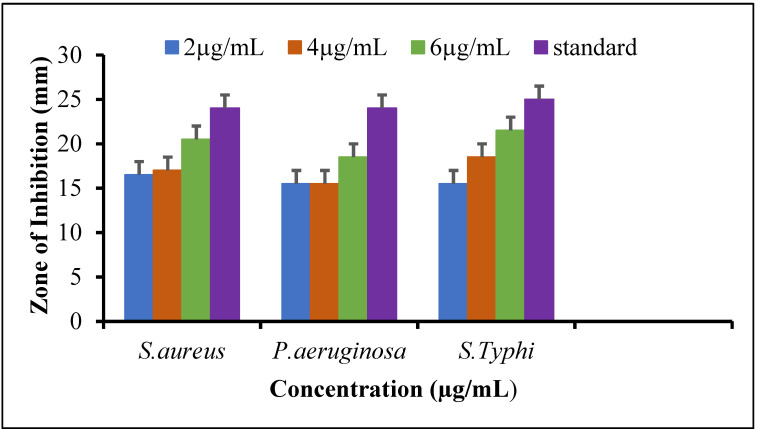
Antibacterial activity of synthesized Ag NPs.

The agar well diffusion assay was employed to perform antibacterial screening. The synthesized Ag NPs that showed antibacterial activity against the tested human pathogens were evaluated at three different concentrations (2 µg/mL, 4 µg/mL, and 6 µg/mL). At each concentration, the Ag NPs demonstrated good antibacterial activity (Figure 5). The Ag NPs exhibited high antibacterial activity against *S. typhi* and *S. aureus* with a 21.5 mm and 20.5 mm zone of inhibition when allied to the standard reference amoxicillin antibiotic with a 25 mm and 24 mm zone of inhibition at concentration of 6 µg/mL, respectively. These findings exhibit that Ag NPs showed an effective zone of inhibition when the concentration of Ag NPs increased. All of the tested bacteria exhibit minimum activity at 2 µg/mL, with a zone of inhibition varying from 11 mm to 18.5 mm. The obtained results confirmed that *S. typhi* exhibit the highest resistance when the concentrations of silver nanoparticles increase. The antibacterial activity of selected bacterial strains increases twofold by increasing the concentration of silver nanoparticles, which revealed the concentration effect on bacterial growth. Moreover, the inhibition zone shown by nanoparticles was comparatively consistent with antibiotics (amoxicillin). This shows that nanoparticles are capable of enhancing the effects of medications. The rationale for using amoxicillin as standard for antibacterial activity was that the Beta-lactam antibiotics such as amoxicillin work by binding proteins and inhibiting certain processes in bacterial cells. This causes the cell walls to break down and destroys the bacteria cell. Therefore, the results conclude that newly synthesized Ag NPs exhibited the same function as the standard drug and showed an excellent efficiency against bacterial strains *S. typhi*, *S. aureus*, and *P. aeruginosa* with increasing concentrations of nanoparticles.

#### 3.6.2. Antifungal Evaluation

**Figure 6:**
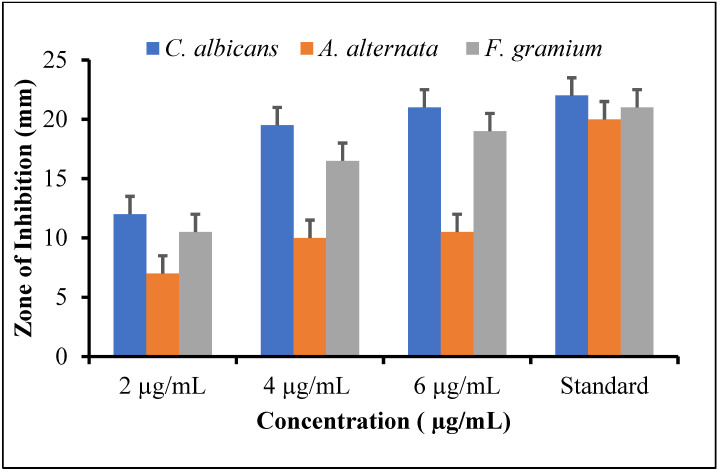
Antifungal activity of synthesized Ag NPs.

To test the antifungal activity of synthesized silver nanoparticles against three fungal strains, *Fusarium gramium*, *Candida albicans*, and *Alternaria alternata*, a standard protocol was employed. Figure 6 showed antifungal activity at concentrations of 2 µg/mL, 4 µg/mL, and 6 μg/mL. The result demonstrated high antifungal activity against *Candida albicans* and *Fusarium gramium* with the zone of inhibition at 21 mm and 19 mm when compared to the standard drug nystatin with a 22 mm and 21 mm zone of inhibition, at 6 µg/mL concentration, respectively. The prepared nanoparticles revealed a remarkable action counter to *Candida albicans*, with an inhibition zone of 21 mm compared to 22 mm for the standard, while *A. alternata* showed moderate activity at other concentrations. This was the first study to evaluate whether Ag NPs incorporated into pathogens enhanced their antifungal property. The antifungal property of the Ag NPs sustained for a longer period. These findings could supplement the treatment protocol of denture-induced stomatitis by reducing the number of pathogens. Although using systemic or topical antifungal drugs such as nystatin is the standard treatment for denture-induced stomatitis, modifying the tissue with Ag NPs might be used as an adjuvant treatment. The rationale for nystatin as standard for antifungal activity was that it binds to ergosterol, a major component of the fungal cell membrane. When present in sufficient concentrations, it forms pores in the membrane that lead to K^+^ leakage, acidification, and death of the fungus.

### 3.7. Discussion

The UV–Vis spectroscopy was employed to characterize nanoparticles, which is consistently proven to be a powerful technique used to analyze nanoparticles. The UV–visible spectroscopy revealed that the reaction of components present in the extracts of plant and an aqueous solution of silver complex reduced the Ag^+^ ions, which demonstrated that Ag NPs in the reaction can be consistent with the spectra of UV–visible analysis. Once the extract of the plant was mixed with an aqueous solution of the silver ion complex, the color transition was detected as a dark yellowish-brown, implying the synthesis of Ag NPs, because of the excitation of surface plasmon vibrations [34]. With the quartz cuvette with distilled water as the reference, the UV–Vis of Ag NPs was recorded with respect to time. The peak broadening in the UV–Vis spectrum specified the poly dispersion of the particles. Within 1 h of the reaction, Ag^+^ ions were reduced to stable nanoparticles, making it one of the fastest green methods for the production of Ag NPs [35]. The SPR band of the Ag NPs solution bears a striking resemblance throughout the reaction to 380 nm, implying that Ag NPs are polydispersed in a colloidal solution with no signs of agglomeration. The nanoparticle solution showed no evidence of agglomeration and remained stable for more than six months [36]. The maximum absorption of synthesized silver nanoparticles was observed at their characteristic wavelength of 440 nm. A similar occurrence was also previously reported in the literature [37]. Furthermore, a different environment parameter was used to examine the synthesis of Ag NPs. A time measurement of Ag NPs was examined by using 0.5 M AgNO_3_ and 5% aqueous plant to optimize the reaction time. Figure 1 shows the ultraviolet absorption spectra of Ag NPs synthesized at various times. It was observed that as the reaction time progresses, the surface plasmon resonance peak intensity increases, and within 15 min, a significant SPR peak intensity is attained. However, after 15 min, the intensity of the SPR band had barely changed. The best time for converting silver ion to silver nanoparticles was 15 min. The effect of the concentration was identified with peak intensity that remarkably increases with increasing AgNO_3_ concentration, and the high SPR band intensity indicates increasing nanoparticle concentration. However, when the AgNO_3_ concentration is increased beyond 0.5 M, the SPR peak intensity slightly decreases, resulting in a broad peak of SPR with maximum wavelengths. The rapid growth of particles at high concentrations could explain this phenomenon. The reactions were then carried out under the above-mentioned conditions, with 0.5 M AgNO_3_ solution used to achieve controlled growth with small particle size. Under given reaction conditions, the effect of volume on Ag NPs synthesis was examined, and the findings revealed as the volume of the reaction is increased, the characteristic SPR absorption peak intensity of Ag NPs also increases. At a volume ratio of 2:5 (*v*/*v*), the maximum absorption was encountered. As a result, the size of Ag nanoparticles increases as the volume increases [38]. The obtained results were similar to our previously reported literature, in which the reduction of silver nanoparticles was achieved by using *Sanvitalia procumbens* plant extract under different factors (such as the concentration of silver salt, volume, temperature, pH and reaction time) [3].

The crystalline nature and morphological determination of nanoparticles was accomplished by using XRD analysis. In this research work, the XRD analysis of silver nanoparticles revealed four distinct diffraction peaks at 2θ positions of 38°, 45°, 65°, and 78°, which correspond to (111), (200), (220), and (311), respectively, with an FCC geometry of silver nanoparticles [39]. The presence of additional and unassigned peaks towards the Bragg peak reflects the FCC geometry of Ag nanocrystals and suggests that bio-organic phase crystallization occurs on the surface of Ag NPs [40]. The small particle size is primarily responsible for the line broadening of the peaks.

**Figure 7:**
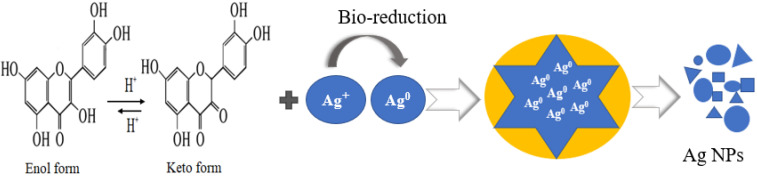
Proposed mechanism for the synthesis of Ag NPs.

The unprocessed silver nitrate performs its deadly work by punching holes in bacterial membranes and wreaking havoc once inside. It binds to essential cell components such as DNA, preventing the bacteria from performing even their most basic functions. Therefore, researchers have focused their attention on synthesizing silver nanoparticles for antimicrobial activity, which showed non-toxic effects. Hence, the synthesized silver nanoparticles exhibited substantial antimicrobial properties against sampling pathogens of human. At 6 µg/mL concentration, the nanoparticles had significant antibacterial activity against *S. typhi* and *S. aureus* with 21.5 mm and 20.5 mm zones of inhibition, respectively, when compared to the standard drug. At lower concentration, all of the test bacteria showed moderate activity, with inhibition zones ranging from 11 to 16.5 mm. Even at high Ag NPs concentrations, *S. aureus* displayed maximum resistance. Likewise, *Trianthema decandra* nanoparticles have been found to be effective against clinically isolated pathogens of human, including *Pseudomonas aeruginosa* and *E. coli* [41]. The antifungal activity of silver nanoparticles was examined against three fungal strains, *Fusarium gramium*, *Alternaria alternata*, and *Candida albicans.* The synthesized silver nanoparticles exhibited remarkable activity against *C. albicans* and *F. gramium*, with inhibition zones of 21 mm and 19 mm compared to the standard, while *A. alternata* showed moderate activity at 6 µg/mL. These findings are consistent with previously obtained Ag NPs by using *Curvularia pallescens*, which revealed that Ag^+^ and Ag NPs showed good efficient against *Cladosporium fulvum* [42]. Lamsal et al. reported that Ag NPs demonstrated, both in vivo and in vitro, the ability to inhibit the growth of *Colletotrichum* species. Because of their high concentration in the solution, silver nanoparticles have a high antifungal activity, which allows them to saturate and adhere to hyphae [43]. Similarly, the synthesis of Ag NPs with a diameter in the range 58–458 nm was carried out using *Mukia maderaspatana* leaf extract. The antimicrobial activity against human pathogens such as *Staphylococcus typhi*, *Bacillus subtilis*, *Staphylococcus aureus*, and *Klebsiella pneumoniae*. The pathogen inhibition efficiency of the synthesized nanoparticle and antibiotic ceftriaxone was compared to that of the free nanoparticle and antibiotic. In comparison to other Ag NPs, those conjugated with ceftriaxone showed the highest inhibition activity [44]. In addition, *Salacia chinensis* extract has been used for the synthesis of Ag NPs and tests their antimicrobial activity against *E. coli*, *Salmonella typhi*, *S. coli*, *Pseudomonas aeruginosa*, and *Staphylococcus aureus*. The antimicrobial activity investigations showed that the *Salacia chinensis* extract-mediated Ag NPs inhibit *P. aeruginosa* and *S. aureus* to the greatest extent [45]. The plant of *Trigonella incise* contained various phytochemicals, such as trigonelline, alkaloids, flavonoids, tannin, saponins glycosides, and phenolic compounds, which serve as capping and reducing agents [22]. However, there is no proper literature that explains how flavonoid reduction and stabilization of Ag NPs work. The extensive studies revealed that -OH groups of flavonoids may have the ability to reduce Ag^+^ to Ag^o^. The tautomeric conversion of flavonoids from enol to keto may release electrons with the liberation of protons in an intramolecular fashion, which helps in the reduction of Ag^+^ to Ag^0^. The proposed mechanism for the synthesis of Ag NPs is illustrated in Figure 7. The findings suggested that flavonoids (-OH) groups could be involved in the reduction of metal ions. *Trigonella incise* is rich in flavonoids with hydroxyl and ketonic groups. When flavonoids react with Ag^+^ as an acid, the most reactive hydroxyl groups attached to the carbon atoms of aromatic ring release electrons that can reduce Ag^+^ to Ag NPs and provide stability against agglomeration. The transfer of charge in both Ag^+^ and flavonoids, the enzymes in plant extracts assemble with Ag^+^ to form an enzyme substrate complex, leading to the synthesis of Ag NPs. Figure 7 depicts a schematic illustration for Ag NPs synthesis from *Trigonella incise* extract, which demonstrates high absorption and enhanced antimicrobial properties. Such a mechanism has been previously reported for the synthesis of Ag NPs. Thus, the synthesis of metal NPs by using plant extract has many more benefits as compared to the chemical route. The plant extract plays a dual role as a reducing as well as a stabilizing agent [46,47].

## 4. Conclusions

In the current research, an aqueous plant extract was used as a stabilizing and reducing agent for the synthesis of Ag NPs, which was performed using a green synthesis approach. The UV–visible spectroscopy was used to confirm the synthesis of nanoparticles. The FCC nature of Ag NPs was revealed by using an XRD pattern. The particle shape of Ag NPs was found to be spherical and size were in the range 20–80 nm. Different function groups and physical interactions of macromolecules with silver were confirmed using FT-IR. The synthesized nanoparticles exhibit good antimicrobial potential. However, in vivo studies will explore their potential use against microbial infectious human diseases.

## Data Availability

All data are incorporated in the MS.

## Sample Availability

Samples can be obtained from Nisar Ahmad.
